# Expiratory aerosol particle escape from surgical masks due to imperfect sealing

**DOI:** 10.1038/s41598-021-91487-7

**Published:** 2021-06-08

**Authors:** Christopher D. Cappa, Sima Asadi, Santiago Barreda, Anthony S. Wexler, Nicole M. Bouvier, William D. Ristenpart

**Affiliations:** 1 Department of Civil and Environmental Engineering, University of California Davis, 1 Shields Ave., Davis, CA 95616 USA; 2 Department of Chemical Engineering, University of California Davis, 1 Shields Ave., Davis, CA 95616 USA; 3 Department of Linguistics, University of California Davis, 1 Shields Ave., Davis, CA 95616 USA; 4 Department of Mechanical and Aerospace Engineering, University of California Davis, 1 Shields Ave., Davis, CA 95616 USA; 5 Air Quality Research Center, University of California Davis, 1 Shields Ave., Davis, CA 95616 USA; 6 Department of Land, Air and Water Resources, University of California Davis, 1 Shields Ave., Davis, CA 95616 USA; 7 Department of Medicine, Division of Infectious Diseases, Icahn School of Medicine at Mount Sinai, 1 Gustave Levy Place, New York, NY 10029 USA; 8 Department Microbiology, Icahn School of Medicine at Mount Sinai, 1 Gustave Levy Place, New York, NY 10029 USA; 9 Present Address: Department of Chemical Engineering, Massachusetts Institute of Technology, 77 Massachusetts Av., Cambridge, MA 02139 USA

**Keywords:** Health care, Engineering

## Abstract

Wearing surgical masks or other similar face coverings can reduce the emission of expiratory particles produced via breathing, talking, coughing, or sneezing. Although it is well established that some fraction of the expiratory airflow leaks around the edges of the mask, it is unclear how these leakage airflows affect the overall efficiency with which masks block emission of expiratory aerosol particles. Here, we show experimentally that the aerosol particle concentrations in the leakage airflows around a surgical mask are reduced compared to no mask wearing, with the magnitude of reduction dependent on the direction of escape (out the top, the sides, or the bottom). Because the actual leakage flowrate in each direction is difficult to measure, we use a Monte Carlo approach to estimate flow-corrected particle emission rates for particles having diameters in the range 0.5–20 μm. in all orientations. From these, we derive a flow-weighted overall number-based particle removal efficiency for the mask. The overall mask efficiency, accounting both for air that passes through the mask and for leakage flows, is reduced compared to the through-mask filtration efficiency, from 93 to 70% for talking, but from only 94–90% for coughing. These results demonstrate that leakage flows due to imperfect sealing do decrease mask efficiencies for reducing emission of expiratory particles, but even with such leakage surgical masks provide substantial control.

## Introduction

Face coverings, such as surgical masks, can reduce the concentration of ambient particles inhaled by a person^1^, while simultaneously decreasing the number of expiratory particles emitted by that person to the surrounding environment^2^. Two factors play a primary role in determining the overall particle reduction efficiency of a given mask type: the material filtering efficiency, which depends on the particle size, and the mask fit, which determines the fraction of air inhaled or exhaled air that passes through the mask versus around the sides of the mask. Overall mask efficiency for inhalation is readily determined using standard mask-fit testing methods, such as those designated by the US Occupational Safety and Health Administration^3,4^. For masks having high material filtering efficiency, such as N95 respirators, leakage around the face seal provides the primary source of ambient particles that are inhaled^3,5^. In contrast, overall mask efficiency for outward emission of expiratory particles is less well established, especially in studies that account for person-to-person variability. Schlieren optical images qualitatively demonstrate that mask wearing substantially redirects the airflow, with much air escaping around the mask rather than passing through it^6–8^. The expiratory aerosol particle concentrations in these leakage flows, however, have not been characterized to date.

The idea that masks can reduce the emission of expiratory particles has a long history^9^, with mask wearing in medical environments becoming common after the influenza pandemic of the early twentieth century. Material filtration tests demonstrate that particle concentrations are reduced, to varying extents, when they pass through filter media^10,11^. Yet, there has been substantial variability in the literature regarding whether mask wearing—as distinguished from wearing of high-efficiency, properly fit respirators—in various environments reduces, for example, the prevalence of surgical site infections^12^ or disease transmission^13,14^. In general, more process-based studies in which external factors are controlled to isolate the effect of mask wearing tend to point to masks providing substantial reduction of emitted microbial or virus containing particles^15,16^. Yet, epidemiological and clinical studies assessing mask effectiveness have provided more ambiguous evidence^17–25^.

In light of the now-common wearing of masks outside of medical contexts and as public health tools owing to the COVID-19 pandemic^26–28^, there is a need to further understand the overall efficacy of masks for reducing expiratory particle emission when worn by people, and in particular to clarify how leakage of air out the sides of masks influences their efficiency. Here, we examine the overall efficiency of surgical masks for reducing expiratory particles in the diameter range 0.5–20 microns produced from talking and coughing when worn by people and demonstrate a method for disentangling the effects of through-mask filtration versus leakage.

## Methods

### Human subjects

**Figure 1:**
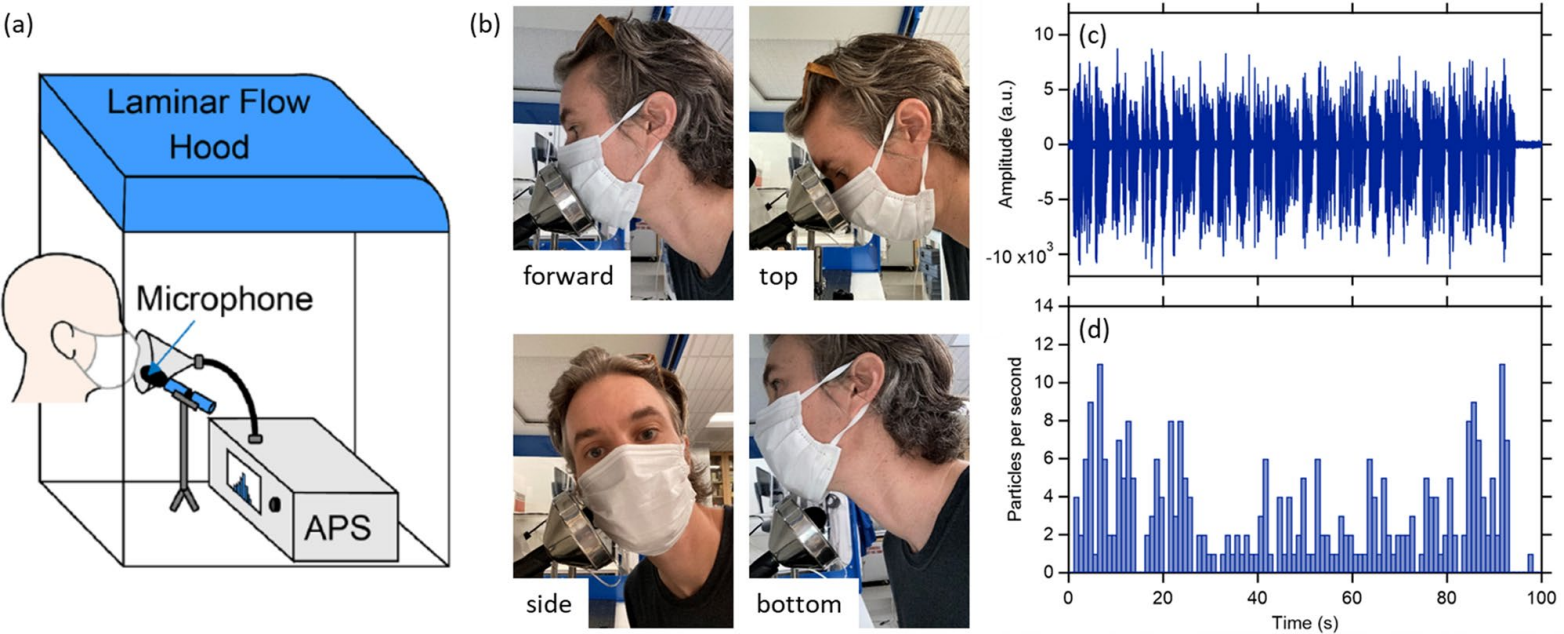
(**a**) Illustration of the experimental setup showing a participant wearing a surgical mask in the through-mask (forward) orientation. (**b**) Photographs of the participant position for the four different sampling orientations: forward, top, side, and bottom sampling. (**c**) Example microphone recording for participant M1 while speaking the rainbow passage with no mask, and (**d**) the associated particle counts by the APS.

We recruited 12 volunteers (seven self-identified male and five self-identified female), ranging in age from 18 to 45 years old. This study was approved by the University of California Davis Institutional Review Board (IRB# 844369-4). All research was performed in accordance with the Institutional Review Board guidelines and regulations. We obtained written informed consent from all participants prior to the tests. Information collected from participants included their age, general health status, and smoking history. Only self-reported healthy non-smokers were included in the study. Informed consent for publication of identifying information was obtained from the participant shown in Fig. 1.

### Experimental setup

The general experimental setup used was similar to that in previous work^2,29,30^. In brief, participants performed various particle-emitting expiratory activities (talking or coughing) in front of the inlet to an aerodynamic particle sizer (APS, TSI Model 3321) placed in a HEPA-filtered laminar flow hood while either wearing or not wearing a standard surgical mask (“Fitty Silky Touch” medical surgical mask, Yuchuan Sanitary Products, Medical Device Registration Certificate Number: 20202140026). Participants were asked to use their fingers to form the metal bar in the surgical mask around their nose. The APS characterizes particles from 0.3 to 20 microns in aerodynamic diameter, with a decreased detection efficiency for particles < 0.5 microns; as such, size distributions are only reported for particles > 0.5 microns. The major difference from the methods described by Asadi et al.^2^ was that the participants here performed the expiratory activities with their head positioned in multiple orientations in front of the APS. In Asadi et al.^2^, all experiments occurred with the participants directly facing the funnel connected to the APS inlet, whether wearing a mask or not. In this forward position, the APS primarily sampled expiratory air that had passed through the surgical mask. Here, in addition to this straight-on “forward” position, particle emission during talking or coughing was characterized with participants in three additional orientations (Fig. 1). The order of sampling in these different orientations was varied randomly between participants. In all cases, the distance between the mask and the plane of the funnel was about 1 cm. Specifically, the four orientations were as follows.

1. *Forward* The participants positioned their head such that the mouth was aimed directly at the center of the APS funnel. This was the orientation examined in prior studies and also referred to as the "through-mask" orientation^2,29,30^.
2. *Top* The participants tilted their heads downward such that the top of the surgical mask, near the bridge of the nose, was approximately centered on the APS funnel; the mouth and nostrils were below the edge of the funnel.
3. *Side* The participants turned their head 90° such that they faced perpendicular to the APS funnel, with the side of the surgical mask approximately centered on the funnel
4. *Bottom* The participants sat higher in the chair such that their mouth was above the edge of the APS funnel and the bottom of the mask, near the chin, was approximately centered on the funnel. For talking, sampling in this position occurred for all participants, whereas for coughing sampling occurred from only three participants but with one participant carrying out six independent replicates; this same participant also repeated the coughing activity in the other orientations.

Observation of particle concentrations exceeding the no mask case while the participant wears a mask can result from shedding of mask fibers or skin^2,31,32^. Such particles are not expiratory particles, but it is still possible that they carry pathogens and thus serve as aerosolized fomites^33^. The average air velocity of the laminar flow hood (0.45 m/s) is comparable to the air velocity for talking (ca. 0.5–1 m/s) but smaller than for coughing (ca. 5–50 m/s). The clean airflow could affect the sampling by the APS by opposing or disrupting the expiratory airflows, more likely for talking as the air velocities are similar and with a potentially even larger influence with mask wearing when the flow is split in multiple directions. The measured particle concentrations from talking measured here agree well with both historical^34^ and recent^35,36^ measurements, indicating minimal impact of the clean airflow. Further, we have observed that measured particle concentrations from speaking remain constant so long as participants remain within ~ 3 cm of the sampling funnel, which was the case for all experiments here. An additional concern is that not all particles are captured from a given orientation owing to flow escaping from directions not sampled. In either case (flow disruption, or directional under sampling), the overall efficacy would be overestimated.

### Expiratory activities

Participants were asked to complete two distinct activities in each orientation.

1. *Talking* Reading aloud the Rainbow Passage, a standard 330-word long linguistic text with a wide range of phonemes. Participants read this passage aloud at an intermediate, comfortable voice loudness. The duration of vocalization (*t*_voc_), excluding the pauses, was determined from the microphone recordings to account for differences in reading pace. The measured particle emission rate, uncorrected for dilution or excess flow, ${\dot{N}}_{p,talk}^{obs}$, was calculated as the total number of particles counted by the APS over the entire (approximately 100-s) reading, divided by *t*_voc_.
2. *Coughing* Successive, forced coughing for 30 s at a comfortable rate and intensity for the participant. The cumulative duration of coughing (*t*_cough_), excluding the pauses between the coughs, was determined from the microphone recordings. The measured particle emission rate, uncorrected for dilution or excess flow, ${\dot{N}}_{p,cough}^{obs}$, was calculated as the total number of particles counted by the APS during the 30 s of measurement, divided by *t*_cough_. One participant (M1) repeated the coughing activity six times.

### Correction of ${\dot{{\varvec{N}}}}_{{\varvec{p}}}^{{\varvec{o}}{\varvec{b}}{\varvec{s}}}$ for flowrate

We define the total time-averaged airflow rate from an expiratory activity as *Q*_exp,tot_. The value of *Q*_exp,tot_ generally varies with the size of a person, with typical values for talking around 10–13 lpm as determined from reading of the Rainbow Passage; higher values were obtained when people counted numbers or spoke the alphabet^37^. Peak flowrates for coughing vary from about 100–1000 lpm, with time-averaged values ranging from ca. 40–240 lpm^38^. Mask wearing will split this total airflow in multiple directions, with the flowrate in any direction *Q*_exp,x_ = *f*_x_^.^*Q*_exp,tot_, where *x* denotes one of the four possible directions for flow, either forward through the mask or out the top, sides, or bottom, and *f*_x_ is the fraction of air in that direction. The APS samples air at *Q*_APS,tot_ = 5 lpm total, from which *Q*_APS,samp_ = 1 lpm is subsampled for characterization of the particle concentration.

The method for converting the particle concentrations measured by the APS to actual expiratory particle emission rates depends on the magnitude of the expiratory airflow rate. When the airflow rate from the expiratory activity directed into the funnel exceeds the APS total flow (*Q*_exp,x_ > *Q*_APS,tot_), the measured particle concentration is representative of the actual particle concentration in the expired airflow, but ${\dot{N}}_{p,x}^{obs}$ underestimates the true particle emission rates _(_${\dot{N}}_{p,x}$) by the ratio *Q*_exp,x_/*Q*_APS,samp_. However, if the airflow rate is smaller such that *Q*_exp,x_ < *Q*_APS,tot_, either as an intrinsic feature of the expiratory activity or because the total expired airflow is split, dilution of the expiratory airflow with the HEPA-filtered ambient air must be additionally accounted for. In such cases, the particle concentration is underestimated by the ratio *Q*_APS,tot_/*Q*_exp,x_, and ${\dot{N}}_{p,x}^{obs}$ underestimates the true particle emission rates by a constant value, *Q*_APS,tot_/*Q*_APS,samp_ = 5. Given typical airflow rates, for speaking and coughing without a mask ${\dot{N}}_{p}^{obs}$ underestimate the ${\dot{N}}_{p}$ by about a factor of 13 and a factor of 40–240, respectively.

The overall mask efficiency, ${\eta }_{tot}$, is:

$${\eta }_{tot}=1-\frac{\sum {\dot{N}}_{p,x}}{{\dot{N}}_{p,nomask}}$$

Quantitative measurements of how surgical masks redirect airflow are lacking, and consequently the *Q*_exp,x_ for each direction are not known a priori.

To address the ambiguity in knowledge of the *f*_x_ and *Q*_exp,x_ values with mask wearing, we used various Monte Carlo-like techniques to investigate how overall mask efficiency depends on the splitting of the airflow. We examined two different approaches: a fully random flowrate approach and a constrained minimum flowrate approach. In the first approach, we assumed random values for all *f*_x_ (with the constraint that the sum equals unity) and calculated the probability distribution of ${\eta }_{tot}$ values for 50,000 iterations; these are referred to as the random flow simulations. In the second approach, we applied a minimum flowrate constraint in each direction (through, top, sides, bottom), with this minimum flowrate (${Q}_{exp,x}^{min}$) set to ensure that the flow-corrected particle concentration in that direction is less than or equal to that determined for no mask. With these constraints the maximum flowrate in any direction equals ${Q}_{exp,x}^{max}={Q}_{exp,tot}-\sum {Q}_{exp,x}^{min}+{Q}_{exp,x}^{min}$.

Four simulations were carried out (with 50,000 iterations), one for each flow direction. The flowrate for the direction of interest was randomly selected from the range ${Q}_{exp,x}^{min}$ to ${Q}_{exp}^{max}$ and the flowrates in the remaining directions were randomly set to be greater than their respective ${Q}_{exp,x}^{min}$ but with their sum equal to the remaining flow. We refer to these simulations as the “random *x* flow” simulations, where *x* is the direction of interest. This second approach allows for exploration of the sensitivity of the results to variation in a particular flow direction.

Since the airflow rates for talking and coughing vary between individuals, we also allowed these to vary in the simulations. Gupta et al.^37^ showed that ${Q}_{exp,tot}^{talk}$ varies with the body surface area (BSA) of the speaker. We have convolved the ${Q}_{exp,tot}^{talk}$-BSA relationship with BSA distributions for males and females using the distribution spreads from Sacco et al.^39^ and population average values from 20 to 79 year olds from Georgiev^40^, assuming 60% males and 40% females (the population of our participants), to determine a probability distribution for ${Q}_{exp,tot}^{talk}$. The resulting distribution is well-described by two Gaussians, the first with a mode of 10.2 lpm and a standard deviation of 1.75 lpm and the second having a mode of 12.4 lpm and a standard deviation of 1.80 lpm. For coughing, the ${Q}_{exp,tot}^{cough}$ was randomly selected from a Gaussian distribution centered at 120 lpm with a standard deviation of 40 lpm, but constrained to be greater than the sum of the minimum flow rates; as will be shown below, the results for coughing are relatively insensitive to the particular choice for the total airflow rate.

### Analysis and statistics

All data processing analyses were carried out using Igor Pro (v. 8.0.4.2, Wavemetrics). Differences between the ${\dot{N}}_{p,x}^{obs}$ values are calculated after log-transformation using a single factor ANOVA test.

## Results

### APS measurements

**Figure 2:**
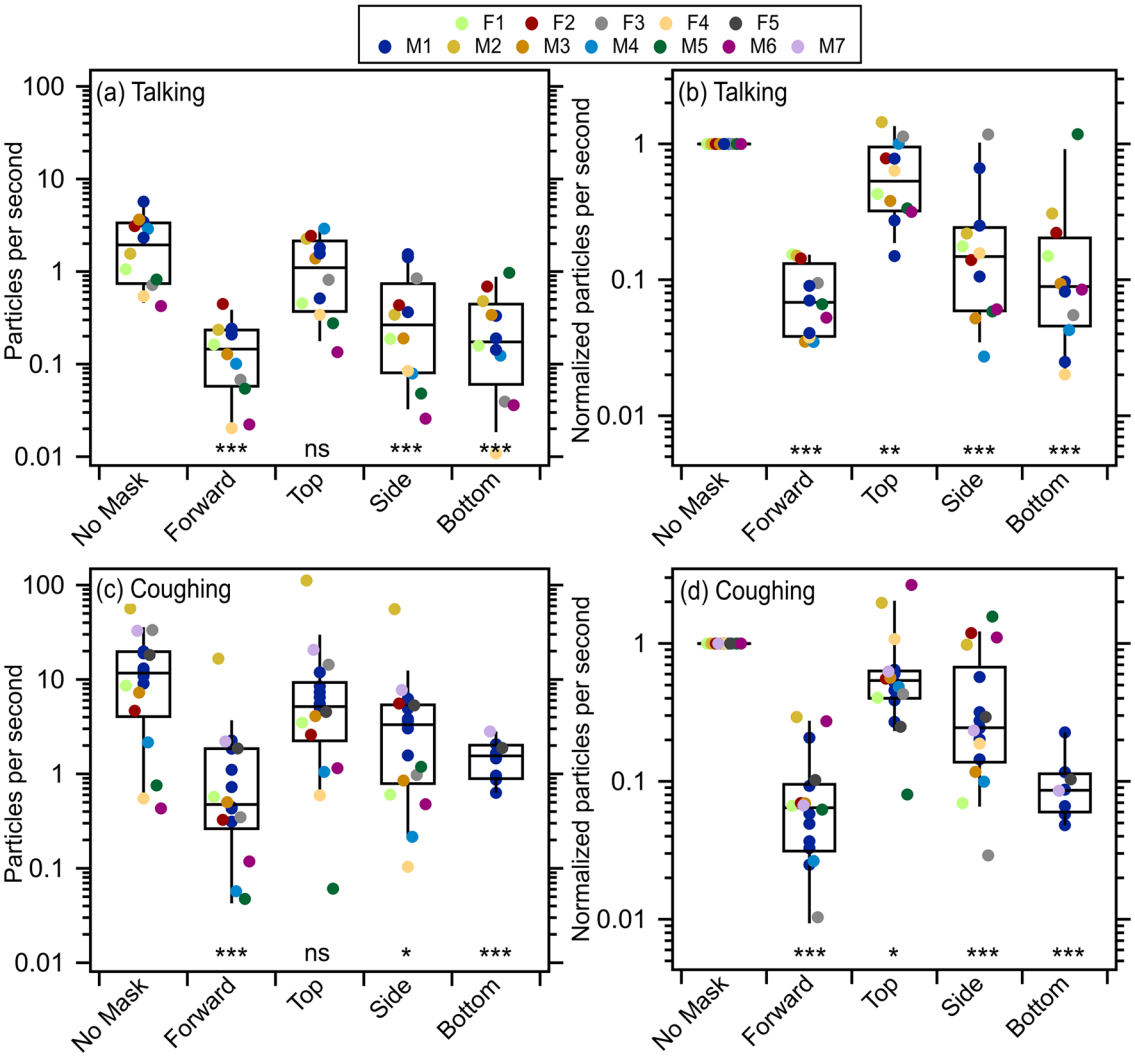
Measured particle count rates, uncorrected for flow, associated with (**a**) talking and (**c**) coughing when participants were positioned in one of the four orientations (forward, top, side, bottom) when masked and with no mask in the forward direction. Measured particle count rates normalized to the no mask case for each person for (**b**) talking and (**d**) coughing. The statistical significance of the difference from no mask is indicated, with ns (*p* > 0.05), * (*p* ≤ 0.05), ** (*p* ≤ 0.01), and *** (*p* ≤ 0.001).

Consistent with our previous observations of through-mask (forward) efficiency for surgical masks during speech^2^, we observe substantial reduction in the median ${\dot{N}}_{p,through}^{obs}$ compared to that observed for no mask for talking, by 93% (Fig. 2a). We note that people tend to vocalize more loudly while wearing masks^2^. Louder vocalization leads to greater particle emission^29^, which might offset some of the reduction owing to mask wearing. Nonetheless, the overarching new result here is that the median particle concentrations measured in the leakage flows at the top, side, or bottom were always less than the no mask but larger than in the flow passing through the mask (forward) Looking to the top orientation, the median ${\dot{N}}_{p,top}^{obs}$ is only somewhat reduced compared to the no-mask condition, by 47%. While the difference in the absolute ${\dot{N}}_{p,x}^{obs}$ values between the no mask and top orientation is not statistically significant (*p* = 0.104), if the ${\dot{N}}_{p,top}^{obs}$ are normalized by the no-mask values for each participant individually (Fig. 2b), the difference is statistically significant (*p* = 0.003). The observed particle emission rates from the sides and bottom are similar, and are intermediate between the through-mask and top-of-mask results; compared to the no-mask condition, the median side-orientation ${\dot{N}}_{p,side}^{obs}$ is 85% lower while the median bottom-orientation ${\dot{N}}_{p,bottom}^{obs}$ is 91% lower, with the reduction from no mask wearing statistically significant in all cases. The above values are averages across all particle sizes characterized (~ 0.3–20 microns). We emphasize that the above values do not account for flow corrections, which are necessary to assess the overall efficiency; this point will be addressed below.

An important feature of the particle emission data is the tremendous variability between participants. As with our previous work^2,29,30^, we observe substantial person-to-person variability for talking both when participants are or are not wearing masks. The person-to-person variability for talking (characterized by the unitless standard deviation, σ, of the log-transformed ${\dot{N}}_{p,x}^{obs}$ values) was lowest for the no-mask condition (σ = 0.37), slightly greater for the forward-mask (σ = 0.42) and top orientation (σ = 0.43), and greatest for the side (σ = 0.59) and bottom (σ = 0.57) positions. However, when the ${\dot{N}}_{p,x}^{obs}$ for each participant are normalized to their individual no-mask ${\dot{N}}_{p}^{obs}$ the person-to-person variability decreases to σ = 0.24 (forward), σ = 0.29 (top), σ = 0.47 (side), and σ = 0.48 (bottom).

The experimental results for coughing exhibited qualitatively very similar trends. We observed substantial reduction in the median ${\dot{N}}_{p,through}^{obs}$ compared to that observed for no mask for coughing, by 94% (Fig. 2c,d). For the top orientation, the median ${\dot{N}}_{p,top}^{obs}$ is reduced compared to the no-mask condition by only 47%. As with talking, the difference in the absolute ${\dot{N}}_{p,x}^{obs}$ values between the no mask and top orientation for coughing is not statistically significant (*p* = 0.22), but the difference is statistically significant if the ${\dot{N}}_{p,top}^{obs}$ are normalized by the no-mask values for each participant individually (*p* = 0.023). The observed median particle emission rate from the side-orientation, ${\dot{N}}_{p,side}^{obs}$, is 75% lower compared to the no-mask condition while the median ${\dot{N}}_{p,bottom}^{obs}$ for the bottom orientation is 92% lower; both differences are statistically significant. As above, these values do not account for flow corrections.

As with talking, substantial person-to-person variability is also observed for coughing when participants are or are not wearing masks. The person-to-person variability of the absolute ${\dot{N}}_{p,x}^{obs}$ is relatively independent of orientation, with σ = 0.69 for no-mask, σ = 0.73 for the forward-mask orientation, σ = 0.81 for the top-mask orientation, and σ = 0.73 for the side-mask orientation; as participant M1 repeated each activity six times, the average of these was used in calculating the variability, rather than each individual measurement, so as to not skew the statistics towards this individual. When calculated for the individual-normalized ${\dot{N}}_{p,x}^{obs}$ the variability decreased to σ = 0.43 (forward), σ = 0.38 (top), and σ = 0.55 (side).

Participant M1 repeated the coughing activities six times with no mask and in each orientation while wearing a mask. The median normalized particle emission rates for coughing while wearing a mask from the single-participant are statistically indistinguishable from all participants for the through-mask (*p* = 0.62), top (*p* = 0.51), and side (*p* = 0.88) orientations based on a single-factor ANOVA test. Further, the ${\dot{N}}_{p,bottom}^{obs}$ for coughing for the two other participants (F5 and M7) who performed this task are very similar to the median from participant M1. This, coupled with the variability for the normalized ${\dot{N}}_{p,x}^{obs}$ being lower than for the absolute ${\dot{N}}_{p,x}^{obs}$ and the similarity to the talking results, strongly suggests that it is reasonable to assume the change from the no-mask case to the bottom orientation for coughing is generally representative.

Notably, participant M2 acted as a coughing superemitter: he emitted more than an order of magnitude more expiratory particles by coughing compared to the rest of the cohort, regardless of orientation (he was not tested for the “bottom” orientation). This same individual was also observed to be a coughing superemitter during prior work (denoted as participant M6 in Asadi et al.^2^). The previous experiments were performed in May 2020, while the experiments reported here were performed in July 2020, suggesting that intrinsic characteristics associated with this individual give rise to the increased emission rates, rather than a temporary condition of some sort. A similar time invariance was also observed previously with respect to speech superemitters^2^.

**Figure 3:**
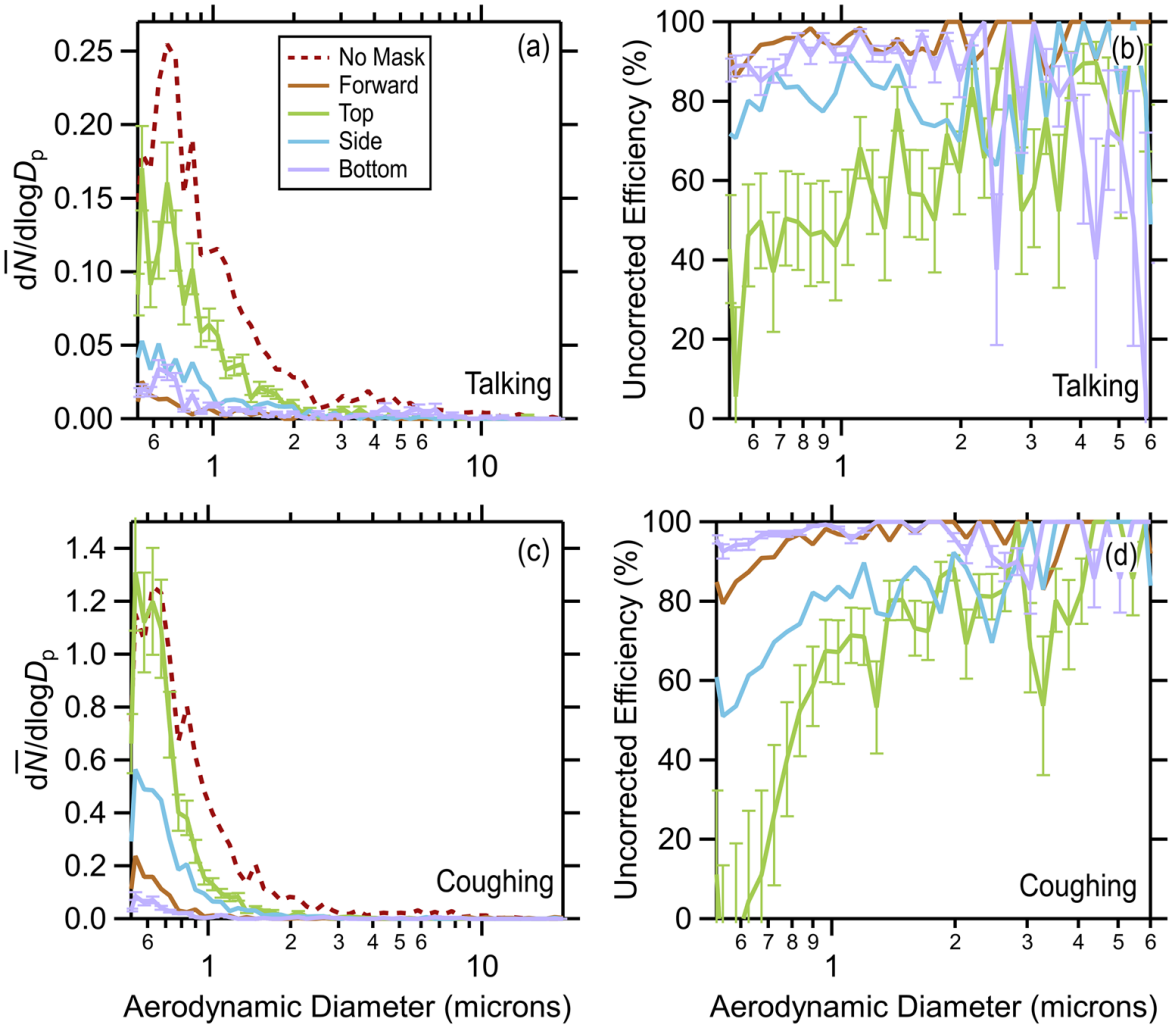
Average particle size distributions observed for no mask and the four orientations (forward, top, side, bottom) when masked for (**a**) talking and (**c**) coughing and the resulting size-dependent particle reduction efficiencies, uncorrected for flow, for (**b**) talking and (**d**) coughing. Error bars are based on repeat (*N* = 10, standard error of the mean) size distribution measurements from one participant for talking made over multiple days, and are shown for the top and bottom orientations, as representative.

Consideration of the average size distributions observed for each position also indicates that for coughing there is a notable size-dependence to the reduction in emitted particles (here, termed the uncorrected efficiency, equal to $1-{\dot{N}}_{p,x}^{obs}/{\dot{N}}_{p,no mask}^{obs}$), most notably for the sampling from the top and side, but also evident in the through position (Fig. 3). The escape of larger (> 1 μm) cough-generated particles from the top or sides of the mask was more efficiently prevented than was the leakage of sub-micron particles in those directions. This observation is consistent with particle loss that is driven by impaction on the mask surface in front of the wearer’s mouth; particle stopping distance and relaxation time increase as the square of particle diameter^41^. For talking, there also appears to be a size dependence to the reduction in emitted particles, although it is less obvious than for coughing. This behavior may reflect the greater noise in the size distributions from talking, but could also result from the much higher air velocity for coughing than talking. For a given particle size, the impaction probability increases with velocity, and will increase the importance of impaction for particles having smaller diameters. Thus, particle impaction could lead to particle removal even for air that does not pass through the mask. A greater role for impaction-driven losses for coughing would be consistent with the finding that the ${\eta }_{tot}$ for coughing exceeds that for talking, discussed below.

### Monte Carlo estimates of overall mask efficacy

**Figure 4:**
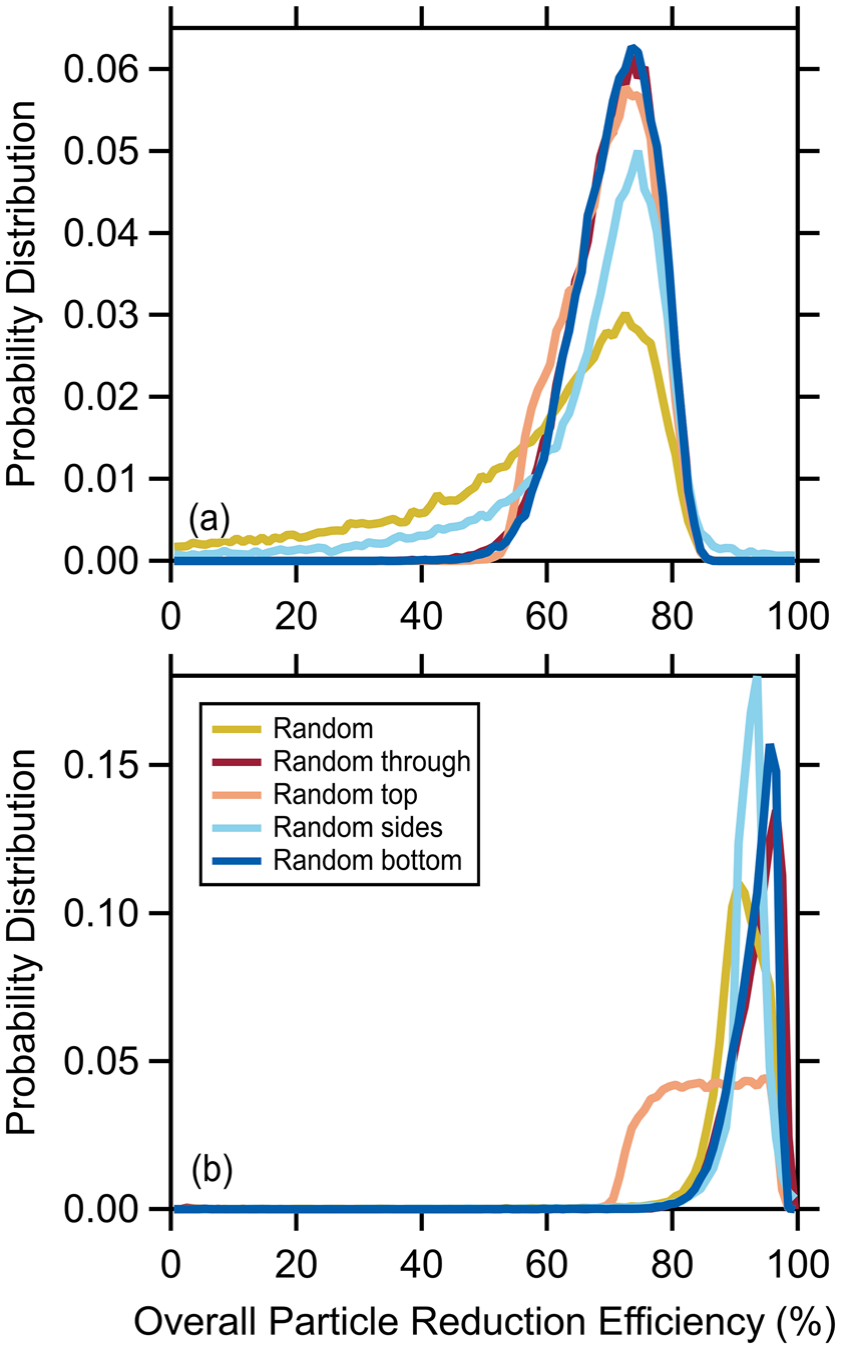
Probability distributions of the particle reduction efficiency for the mask for (**a**) talking and (**b**) coughing, as determined from simulations that use different constraints on the directionality of the expiratory airflow.

**Figure 5:**
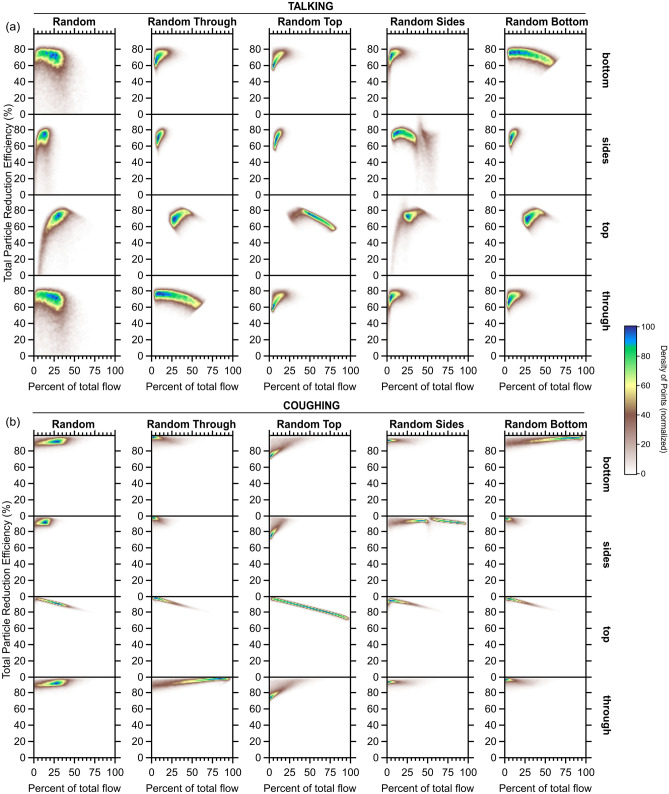
Two dimensional histograms of the calculated total particle reduction efficiency for the mask versus the percent of the total flow that passes through the mask, escapes out the top, escapes out the sides, or escapes out the bottom for simulations with the five differing model constraints on the flows for (**a**) talking or (**b**) coughing. Colors indicate the density of individual results from the simulation iterations, from brown (low density) to blue (high density). The labels at the graph tops (e.g., “Random”) indicate the particular model constraint while the labels on the right (e.g., “bottom”) refer to the direction of the airflow. For example, the graph for the [Random, through] pair shows the probability that a particular particle reduction efficiency is achieved for air having passed through the mask when the flowrates in each direction are randomly chosen.

**Figure 6:**
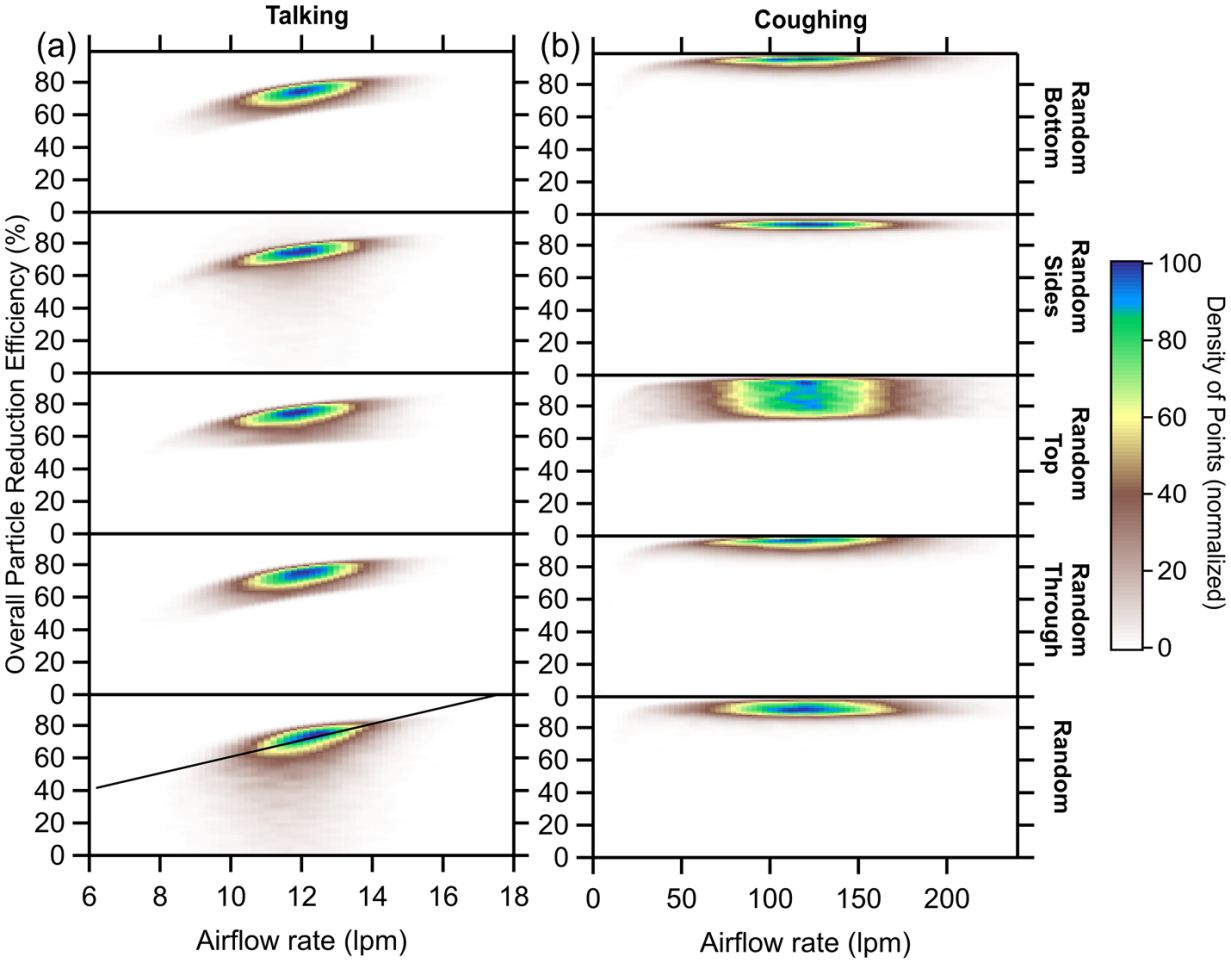
Two-dimensional histograms of the calculated overall particle reduction efficiency for the mask versus the total expiratory airflow rate for (**a**) talking and (**b**) coughing for the five different simulations. Colors indicate the density of individual simulation results (brown = low density, blue = high density).

We estimate the overall mask efficiency for talking and coughing using the various Monte Carlo-like simulations described above to determine flow-corrected particle emission rates in all orientations, from which the flow-weighted overall efficiency derives. For talking, the ${\eta }_{tot}$ probability distributions peak at a similar ${\eta }_{tot}$ for all simulations, around 74%, and fall off sharply towards higher efficiencies (Fig. 4a). The random flow and random side flow ${\eta }_{tot}$ distributions have a relatively long tail towards lower efficiencies, while the distributions are quite narrow for the other simulations. The weighted average ${\eta }_{tot}$ = 64 ± 8% across all five simulations notably decreased from the median (non-flow-corrected) through-mask efficiency (93%). Two-dimensional histograms relating the ${\eta }_{tot}$ to the *f*_x_ values for each simulation provide further insight into the dependence of the overall mask efficiency on splitting of the air expiratory when talking (Fig. 5a). There is generally little dependence of the ${\eta }_{tot}$ on the *f*_through_, *f*_sides_, and *f*_bottom_. The ${\eta }_{tot}$ does exhibit some dependence on *f*_top_, most notably decreasing with increasing *f*_top_ for the random top flow simulation, likely owing to the *f*_top_ reaching their largest values in this simulation. We have also explored the dependence of ${\eta }_{tot}$ on the ${Q}_{exp,tot}$ (Fig. 6a); for talking, the ${\eta }_{tot}$ generally increases with ${Q}_{exp,tot}$ at a rate of about 5% per lpm. Regardless, these simulations indicate that the most probable ${\eta }_{tot}$ for talking with the surgical mask is around 70%. Our observations and analysis demonstrate that expiratory particles do escape the mask, carried by air that passes around the mask rather than through it; however, despite these losses, the overall mask efficiency remains high, compared with respiratory particle emissions in the absence of a mask.

For coughing, the ${\eta }_{tot}$ probability distributions from the various simulations are very similar for all simulations except the random top flow simulation (Figs. 4b, 5, 6). Interestingly, all of the simulations indicate that the ${\eta }_{tot}$ for coughing exceeds that for talking, with a mean across all simulations of 90 ± 3%, only slightly smaller than the (non-flow-corrected) through-mask efficiency (94%) (Fig. 4b). Unlike talking, the ${\eta }_{tot}$ exhibits no dependence on the total expired airflow rate, except for unreasonably low ${Q}_{exp,tot}^{cough}$ (< ~ 30 lpm; Fig. 6b). The ${\eta }_{tot}$ for coughing exhibits a notable sensitivity to the *f*_top_ values for all simulations, most evident for the random top simulation, with ${\eta }_{tot}$ decreasing with *f*_top_ (Fig. 5b). The simulation results indicate that for coughing lower ${\eta }_{tot}$ values are strongly connected to the amount of expiratory airflow that exhausts out the top of the mask, as perhaps expected given the similarity of the ${\dot{N}}_{p,top}^{obs}$ to ${\dot{N}}_{p,no mask}^{obs}$ for coughing. Overall, our results indicate that the overall mask efficiency is greater for coughing than for talking and near 90%.

The actual through-mask efficiency may differ from that indicated by direct comparison of ${\dot{N}}_{p,through}^{obs}$ to ${\dot{N}}_{p,no mask}^{obs}$, as in Fig. 2, owing to the flow-correction of both. The true efficiency associated with each direction will exceed that indicated by comparison of the ${\dot{N}}_{p,x}^{obs}$ values because ${Q}_{exp,x}$ < ${Q}_{exp,tot}$, and thus the flow correction for the no mask observations will exceed that for any of the observations with the participants wearing the mask. We assess the distributions of flow-corrected efficiencies for each direction (${\eta }_{x}$) from the simulations. For both talking and coughing, the flow-corrected ${\eta }_{through}$, ${\eta }_{side}$, and ${\eta }_{bottom}$ probability distributions indicate these efficiencies are all > 90%. The simulations indicate lower values for ${\eta }_{top}$, with most values around 80% for talking and > 80% for coughing (Fig. S1).

**Figure 7:**
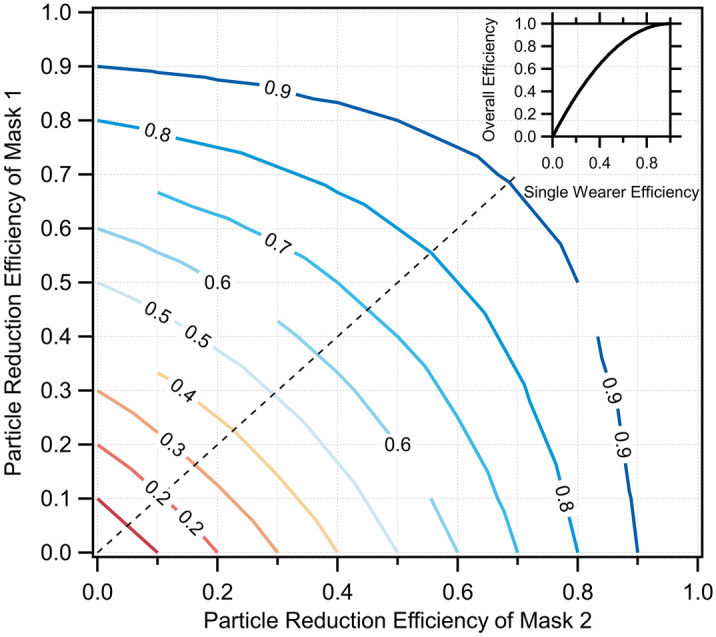
The net particle reduction efficiency with masks worn by source persons and receptor persons, for the wearer masks having varying efficiency. (Inset) The overall reduction efficiency versus single mask reduction efficiency assuming the same mask efficiency for the source person and receptor person.

The above allows for assessment of the combined risk reduction for disease transmission via inhalation of expiratory particles. The net efficiency for simultaneous mask wearing by an infectious source (e.g., the infected person exhaling or coughing a respiratory pathogen into the air) and by a susceptible recipient (i.e., the person subsequently inhaling the pathogen from the air) is given by ${\eta }_{net}=1-(1-{\eta }_{tot,P1})(1-{\eta }_{tot,P2})$. Thus, the net efficiency from source to receptor changes non-linearly with the efficiency of either the source or the receptor (Fig. 7).

## Discussion and conclusions

We examined the effectiveness of surgical masks at reducing emission of expiratory particles produced from talking or coughing after accounting for air escape out the edges of the mask. As previously observed, the particle concentration for air that passes through the mask is reduced substantially from no mask wearing. We find that air escaping out the mask top (by the nose) carries the most particles and provides the smallest reduction compared to no mask wearing. Air escaping out the sides leads to somewhat greater reduction in particles compared to air that escapes out the top. Particle reduction occurs even for air that escapes, which indicates that impaction of particles into the mask surface plays an important role in removing particles from expiratory air. One aspect not considered here, but of potential importance, is the influence that wearing of glasses or goggles by individuals might affect particle escape out the mask top by influencing the extent of sealing between the mask and face. It is possible that wearing glasses or goggles could decrease particle escape and increase the overall mask efficiency.

We estimated the overall mask efficiency for reducing expiratory particle emissions, accounting for air that passes through the mask versus that escapes from the edges. For talking, air escaping reduces the efficiency from > 90% (for air that passes through the mask) to about 70%. For coughing, the reduction in efficiency from air escape is notably less, decreasing only from 94 to 90%. Overall, our observations demonstrate that, while air escape does limit the overall efficiency of surgical masks at reducing expiratory particle emissions, such masks nonetheless provide substantial reduction.

Our results demonstrate that escape of particle-laden air from the top, primarily, but also from the sides and bottom of surgical masks worn by people provides the primary means by which expiratory particles entrain into the local environment. This behavior is driven by the overall high particle removal efficiency for air that passes through the mask, but also by substantial reduction in particles even for air that escapes out the mask edges. Impaction likely drives the reduction in particles in air that escapes, with larger particles lost with greater efficiency than small particles.

Previous observations from our team^2^ and others^42^ of through-mask efficiencies, or of material efficiencies^10,43–46^, are generally consistent with the observations presented here, although we note that some studies have found relatively poor material efficiencies for some surgical masks^10,47^. Our results are also generally consistent with recent unpublished observations examining the material efficiency along with the inward (breathing) and outward (exhaling) particle reduction associated with surgical mask wearing by manikins during simulated breathing^48^. Experiments examining the effectiveness of masking on simulated aerosol emission from coughing using a manikin head yield a somewhat smaller overall efficiency (59%) compared to our observations (90%)^49^. It is possible that this difference results from our experiments having considered mask wearing and coughing by real people, as opposed to simulated coughing using a manikin head, along with potential differences in the aerosol size distributions given the size dependence of the mask efficiency (Fig. 3). Here we tested only standard surgical masks; it appears likely that other types of masks that have been shown to reduce forward particle emission through the mask would also behave similarly in terms of particle emission through the leakage flows, albeit with differences in the extent of air leakage depending upon fit, but direct experimental measurements are necessary to corroborate this hypothesis.

It is of interest to consider how our results might apply to reduction of inhaled particle concentrations, as opposed to expiratory particles. We expect that the through-mask efficiency for inhalation would be similar as for expiration. However, it seems likely that reduced particle removal relative to expiration would occur for particles carried in inhaled air that does not pass through the mask. Whereas expiratory particles are initially directed towards the mask and must turn (in response to air flow) prior to impacting mask if they are to survive to the surrounding environment in the air that escapes, this is not the case for inhaled particles. While the inhaled particles in air that have not passed through the mask must still make a turn to enter a persons’ mouth or nose, there is no mask barrier into which they can impact. As such, it may be that the efficacy of mask wearing to reduce concentrations of inhaled particles exhibits greater sensitivity to the fraction of inhaled air that passes through the mask, which may differ between inhalation and exhalation. This hypothesis seems reasonably consistent with recent measurements^48,50^. However, differences in how a given mask seals during inhalation versus exhalation could further contribute to differences in overall efficiency associated with the two activities.

As a key practical conclusion, our results strongly corroborate the efficacy of surgical masks at significantly reducing emission of expiratory particles into the surrounding air, despite the existence of non-filtered leakage flows around the sides of the mask. In other words, the existence of such leakage flows does not obviate mask wearing; rather, our results confirm that mask wearing provides a significant reduction in the probability of disease transmission via expiratory particles, especially when both the infected and susceptible individuals wear masks. Further, without a mask the expiratory airflow will travel directly away from the talker or cougher in a high velocity plume towards others who may be nearby^51^, whereas with mask wearing the leakage flows are redirected upwards, sideways, and downward leading to more rapid dilution along with a reduction in the velocity of the outward jet^6,52^. Our results indicate that public health authorities should continue to emphasize mask wearing to protect against transmission of COVID-19 and other respiratory diseases.

## Supplementary Information

Supplementary Figure S1.
