# Supplementary material for: Expiratory aerosol particle escape from surgical masks due to imperfect sealing
Source: Sci Rep. 2021 Jun 8;11:12110. doi: 10.1038/s41598-021-91487-7 (PMC8187651; doi:10.1038/s41598-021-91487-7)
Supplement: Supplementary file 1 — Supplementary Figure S1. [file 41598_2021_91487_MOESM1_ESM.pdf]

Supplemental material for "Expiratory aerosol particle escape from surgical masks due to imperfect sealing"

Christopher D. Cappa[1,*], Sima Asadi[2,§], Santiago Barreda[3], Anthony S. Wexler[2,4,5,6], Nicole M. Bouvier[7,8], & William D. Ristenpart[2]

[1] Dept. of Civil and Environmental Engineering, Univ. of California Davis, 1 Shields Ave., Davis, CA 95616 USA
[2] Dept. of Chemical Engineering, Univ. of California Davis, 1 Shields Ave., Davis, CA 95616 USA.
[3] Dept. of Linguistics, Univ. of California Davis, 1 Shields Ave., Davis, CA 95616 USA.
[4] Dept. of Mechanical and Aerospace Engineering, Univ. of California Davis, 1 Shields Ave., Davis, CA 95616 USA.
[5] Air Quality Research Center, Univ. of California Davis, 1 Shields Ave., Davis, CA 95616 USA.
[6] Dept. of Land, Air and Water Resources, Univ. of California Davis, 1 Shields Ave., Davis, California 95616 USA.
[7] Dept. of Medicine, Div. of Infectious Diseases, Icahn School of Medicine at Mount Sinai, 1 Gustave Levy Place, New York, NY 10029 USA.
[8] Dept. Microbiology, Icahn School of Medicine at Mount Sinai, 1 Gustave Levy Place, New York, NY 10029 USA.
[§] Now at: Dept. of Chemical Engineering, Massachusetts Institute of Technology, 77 Massachusetts Av., Cambridge, MA 02139 USA.
*Corresponding author: cdcappa@ucdavis.edu

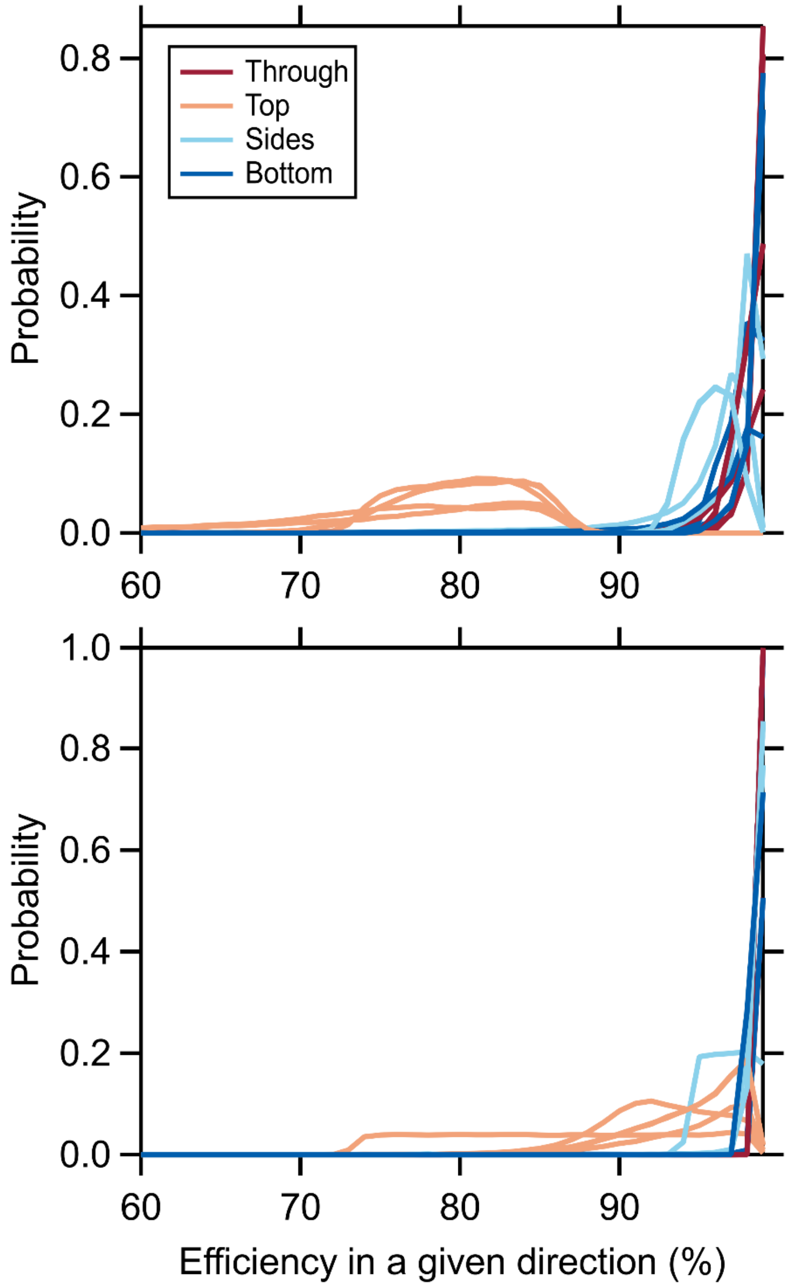

Figure S1. The direction-specific, flow-corrected particle reduction efficiencies derived from each of the five simulations. The lines are colored according to the flow direction, and are not distinguished between the simulations.
